# Treadmill Experience Alters Treadmill Effects on Perceived Visual Motion

**DOI:** 10.1371/journal.pone.0021642

**Published:** 2011-07-25

**Authors:** Yoshiko Yabe, Hama Watanabe, Gentaro Taga

**Affiliations:** 1 Graduate School of Education, University of Tokyo, Tokyo, Japan; 2 Research Institute of Kochi University of Technology, Kochi, Japan; McMaster University, Canada

## Abstract

Information on ongoing body movements can affect the perception of ambiguous visual motion. Previous studies on “treadmill capture” have shown that treadmill walking biases the perception of ambiguous apparent motion in backward direction in accordance with the optic flow during normal walking, and that long-term treadmill experience changes the effect of treadmill capture. To understand the underlying mechanisms for these phenomena, we conducted Experiment 1 with non-treadmill runners and Experiment 2 with treadmill runners. The participants judged the motion direction of the apparent motion stimuli of horizontal gratings in front of their feet under three conditions: walking on a treadmill, standing on a treadmill, and standing on the floor. The non-treadmill runners showed the presence of downward bias only under the walking condition, indicating that ongoing treadmill walking but not the awareness of being on a treadmill biased the visual directional discrimination. In contrast, the treadmill runners showed no downward bias under any of the conditions, indicating that neither ongoing activity nor the awareness of spatial context produced perception bias. This suggests that the long-term repetitive experience of treadmill walking without optic flow induced the formation of a treadmill-specific locomotor-visual linkage to perceive the complex relationship between self and the environment.

## Introduction

Walking on solid ground is linked with its contingent feedback such as backward optic flow. When we walk on a treadmill, we experience altered linkage between self-induced motion and the predicted multisensory feedback. After we have run on a treadmill for short periods on the order of minutes, motor/perceptual aftereffects, called “treadmill illusions,” take place when we run on solid ground [1]–[3]. It was reported that participants who attempted to jog in place on solid ground jogged forward after brief treadmill running [1]–[2]. Other study showed that the participants who were instructed to maintain a constant ‘visual’ speed while repeatedly walking a 5 m course after 20 min of treadmill running were seen to accelerate their pace [3]. These instant aftereffects are thought to reflect multisensory adaptation to the altered linkage during treadmill locomotion. Yabe & Taga [4] reported a different type of treadmill illusion, the phenomenon of “treadmill capture,” in which the participants more frequently judge ambiguous apparent motions shown in front of their feet to be moving downward when they are walking on a treadmill compared with standing on one. It was further shown that the prolonged experience of using treadmills is associated with a reduction in the magnitude of treadmill capture [5]. This suggests that in contrast to the instant aftereffects of treadmill locomotion in a laboratory setting, long-term treadmill exercise, in which users are repeatedly exposed to the process of adaptation and washout, induces slow, gradual adaptive changes in motor-perceptual linkage. However, we do not fully understand the underlying mechanisms for treadmill capture and its adaptive changes induced by the possible changes in motor-perceptual linkage. The aim of the present study is to clarify these issues by examining treadmill capture in the participants without experience in treadmill running and in those with habitual experience in treadmill running.

Treadmill capture has been interpreted to have the function to solve the correspondence problem in the perception of ambiguous visual motion [4]. From the standpoint of von Helmholtz [6], seeing motion is not merely “seeing” a series of retinal images, but an unconscious conclusion (*unbewussten Schlüsse*) about a certain object that was in one place and then another. Given that there are multiple interpretations of point-by-point correspondences between the image in a certain moment and that of its immediate aftermath [7]–[9], the visual system has to solve the correspondence problem to perceive the position shift of external objects in space in real time. When vision and other modalities are stimulated concurrently, the extra-retinal cues work to solve this correspondence problem by constraining the range of the possible interpretation of bidirectional information. For instance, tactile information about the direction of motion influences visual perception [10]. Sounds can alter the visual perception of motion [11], and motor behaviors also contribute as a cue to perceive visual motion [12]–[15]. In particular, Hu and Knill [16] indicated that kinesthetic information generated by active hand movements alters the perceived direction of visual motion. In contrast, Wohlschläger [17] showed that movement preparation is sufficient to bias the perceived direction of visual motion. Thus, we can consider two main possible sources of cues to solve correspondence problem while active movements: ongoing activity and cognitive process. With regard to treadmill capture, although the ongoing activity of walking is likely to bias the perceived direction of visual motion [4], it is unclear whether cognitive process such as awareness of being on a treadmill may also bias the perception. Furthermore, it is an open question how long-term treadmill exercise induces changes in motor-perceptual linkage, although the previous study showed the reduction of the treadmill capture effect through the treadmill experience [5].

**Figure 1:**
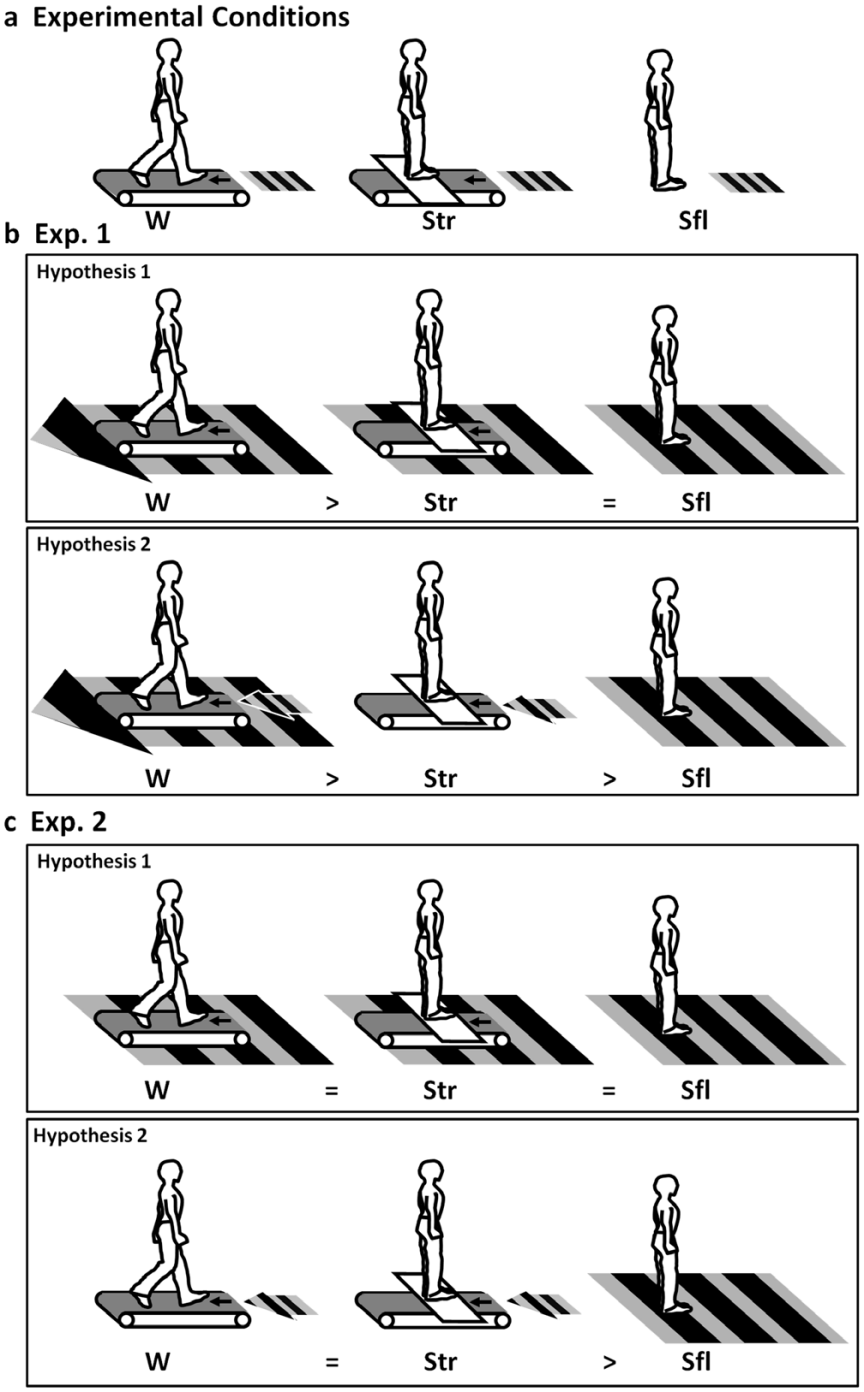
The schematic illustrations of the experimental conditions and hypotheses. (**a**) Experimental conditions, W (left), Str (center), and Sfl (right) show the conditions of walking, standing on a treadmill, and standing on the floor, respectively. The apparent motion stimulus of horizontal gratings is presented in front of the participants' feet in each condition. (**b**) Hypotheses 1 (above) and 2 (below) of Experiment 1. The bold striped arrow indicates the participants' assumption of optic flow of ground surface that produces downward bias to the perceived direction of the stimuli under the condition. The bold striped square indicates the participants' assumption of zero optic flow of ground surface that produces zero bias to the perceived direction of the stimuli. The thin striped arrow indicates the participants' assumption of being on a treadmill, which produces downward bias to the perceived direction of the stimuli. In Hypothesis 1, the downward bias under Condition W is larger than that under the other two standing conditions, Str and Sfl. In Hypothesis 2, the downward bias under Condition W is larger than that under Condition Str. In addition, the downward bias under Condition Str is larger than that under Condition Sfl. (**c**) Hypotheses 1 (above) and 2 (below) of Experiment 2. In Hypothesis 1, there is no downward bias under the three conditions. In Hypothesis 2, the downward biases under Conditions W and Str are larger than that under Condition Sfl.

In the present study, we performed experiments on treadmill capture with non-treadmill runners (nTRs), who had never used a treadmill (Exp. 1), and treadmill runners (TRs), who used a treadmill regularly (Exp. 2). In Yabe & Taga [5], durations of treadmill exercise varied from 2 months to 24 months. In order to control the amount of the treadmill experience of the TR group, we recruited participants who had treadmill exercise for more than 12 months. The participants in each experiment performed two-alternative, forced-choice tasks to judge the motion direction of the apparent motion stimuli of sinusoidal horizontal gratings under three conditions, as shown in Figure 1a. Under the walking (W) condition (Figure 1a, left), the participants viewed the stimuli co-aligned with the extension of a treadmill belt as they walked. Under the standing-on-treadmill (Str) condition (Figure 1a, center), they viewed the same stimuli as they stood on a platform across the moving treadmill. Under the standing-on-floor (Sfl) condition (Figure 1a, right), they viewed the same stimuli as they stood on a platform separate from the treadmill on the floor. Comparing the results of Conditions W and Str allows us to investigate the influence of the ongoing activity of walking on the perceived direction of visual motion. Comparing the results of Conditions Str and Sfl allows us to investigate whether only the difference in spatial context, which probably induces the participants' awareness of being on a treadmill or the floor, affects the perceived direction of visual motion. Note that the awareness we intended to observe here bases on the cognitive information about the context of being on a treadmill. Since the participants moved to the position of each condition, they were aware of whether they were on the treadmill or on the step. To rule out the influence of visual information of such context, all experiments were performed in a completely dark room so that the participants could not use any visual cues other than the stimulus. The treadmill was concealed from participants view with black cloth.

In Exp. 1 with nTRs, we first assumed that the participants perceive downward visual motion more frequently under Condition W than under Condition Str on the basis of previous studies [4], [5]. Then, we proposed two hypotheses. According to Hypothesis 1 (Fig. 1b, above), the ongoing activity of walking on a treadmill but not the awareness of the spatial context of being on a treadmill biases the perceived direction of visual motion as if the backward optic flow is present, probably because of the tight linkage between forward walking on the ground and the backward optic flow regarding the ground surface. This hypothesis predicts that the participants perceive downward motion more frequently under Condition W than under the other two standing conditions Str and Sfl. According to Hypothesis 2 (Figure 1b, below), the ongoing activity of walking on a treadmill biases the perceived direction of visual motion, as in Hypothesis 1. In addition, contextual awareness of being on the treadmill biases the perceived direction of visual motion. This hypothesis predicts that the participants perceive downward motion more frequently under Condition W than under Condition Str, and that they perceive downward motion more frequently under Condition Str than under Condition Sfl. In Exp. 2 with TRs, we assumed that there was no difference between Conditions W and Str on the basis of a previous study on the reduction of the treadmill capture effect in habitual treadmill runners [5]. Then, we have two hypotheses. According to Hypothesis 1 (Figure 1c, above), neither the ongoing activity of walking on a treadmill nor the contextual awareness of being on one biases the perceived direction of visual motion, probably because of the adaptation to the linkage between treadmill walking and no self-motion in reference to the ground, which should produce no backward optic flow of the ground surface. This hypothesis predicts that there are no biases in visual motion perception under all three conditions. According to Hypothesis 2 (Figure 1c, below), contextual adaptation to being on a treadmill, irrespective of the ongoing activity of walking or standing, biases the perceived direction of visual motion, probably because of the expectation of the backward optic flow of the motion of the treadmill belt. On the other hand, there is no perceived bias under Condition Sfl.

## Experiment 1

### Methods

#### Ethics Statement

Ethical approval was obtained for this study from the ethical committee of the Graduate School of Education, University of Tokyo, and written informed consent was obtained from all participants prior to the initiation of the experiments.

#### Participants

Twenty-two healthy adults who had never used a treadmill (nTRs; 12 males and 10 females; mean age 21.6 years) participated in Exp. 1. They were all naive to the purpose of the study. Each participant had normal or corrected-to-normal vision. We limited the height of the participants to 180 cm because the walkable area of the treadmill, which was narrowed in the experimental settings, was not long enough for the participants taller than 180 cm. Data from three participants were excluded from the analysis because these participants reported that they had not fixated on the stimulus. We further excluded the data from a participant whose R-squared, the contribution ratio of the psychometric function, was very low only under Condition Sfl (0.06) because it is suspected to be inappropriate in the arousal level.

#### Apparatus

**Figure 2:**
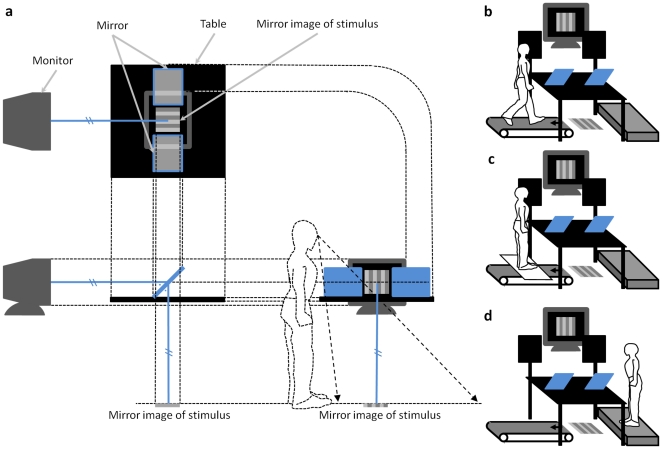
Experimental setup. (**a**) Orthographic drawing of top view (top left), front view (bottom left), and right-side view (bottom right) of the monitor, the table, and the mirrors (depicted in blue). Each mirror was arranged to show the stimulus in front of the observer's feet. The light ray from the CRT monitor is drawn as yellow solid lines. The line of sight to the mirror image is drawn as yellow dashed lines. (**b**) Condition W. (**c**) Condition Str. (**d**) Condition Sfl.

Figure 2a presents an illustration of the experimental setup. The trials of Conditions W (Figure 2b) and Str (Figure 2c) were performed on a treadmill (NIHON KOHDEN Aeromill STM-1420). The trials of Condition Sfl (Figure 2d) were performed on a platform, which was adjusted to have the same height above the floor as that of the surface of the treadmill belt. A mirror was placed on a table in front of each position where the participant walked or stood. All stimuli, presented on a cathode ray tube (CRT) monitor (EIZO T961), were viewed through the mirror. The position and tilt of each mirror were adjusted for each participant so that the reflection of the display seen in the mirror appeared to occupy the position on the ground surface in front of the participant. The table and the front part of the treadmill apparatus were covered with black felt so as not to provide inappropriate visual cues. Blackboards obstructed the direct view of the CRT monitor. Stimuli were generated using OpenGL on a Windows computer and were presented at a refresh rate of 60 Hz and a resolution of 1024×768 pixels.

#### Procedure

The experiments were conducted in a completely dark room. Illuminance obtained on the treadmill belt was 0.09 lx when the visual stimulus was presented. The participants could not see the treadmill belt, because it was obstructed by the table on which the mirror was placed (Figure 2). Under Condition W, the participants walked on a treadmill (Figure 2b). They used the handrails for safety purposes and not to let their arms bear their weight. Under Condition Str, they stood on a board bridged over the treadmill (Figure 2c). Under Condition Sfl, they stood on a platform (Figure 2d). Under the two standing conditions, the participants were told not to move their limbs as much as possible. Under all conditions, the participants looked into the mirror in front of them to see visual stimuli projected in front of their feet. Under all conditions, the treadmill ran at a speed of 91.7 cm/s.

Each participant determined the position on the treadmill at which he or she could stand or walk throughout the experiment in a stable condition. In each trial, apparent motion stimuli of a horizontal grating pattern were presented for 3 s. The participants were instructed to view the entire stimuli of horizontal gratings and not to fixate on its outline. They were told that “the gratings move upward or downward.” The stimulus was followed by a 10 s presentation of a central white cross on a black background as a fixation target for gazing. As soon as the display switched from the stimulus to the screen of the fixation cross, the participants orally reported whether the motion direction of the gratings presented just prior was downward or upward. All responses were entered into a computer by an experimenter without any feedback.

**Figure 3:**
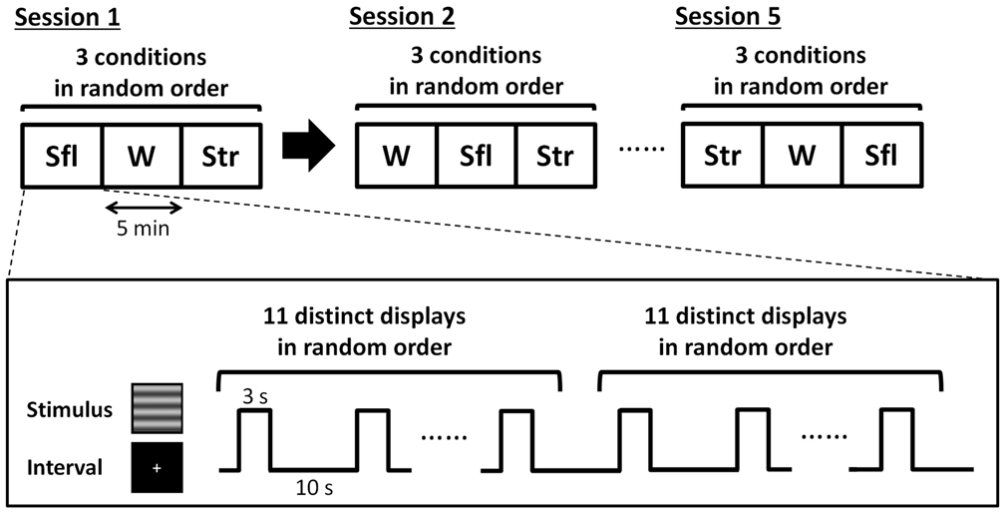
Procedure. Upper part represents the design of the sessions. The square with ‘W’, ‘Str’, and ‘Sfl’ represents the block of Condition W, Str, and Sfl, respectively. The order of the three conditions was randomized in every session. They took rest for about 6 min between sessions. Lower part represents the design of a block. Each block which takes approximately 5 min consisted of 22 trials and presented 11 distinct stimulus displays shown two times in a random order. Participant reported the direction of motion during interval (10 s) following stimulus display (3 s).

Each participant took part in five experimental sessions after a brief practice session (see Figure 3). One experimental session consisted of three blocks of each condition in a random order. Each block consisted of 22 trials and represented 11 distinct stimulus displays shown two times in a random order. The total amount of time required for an experiment was approximately 160 min.

#### Stimuli

The stimuli, which were essentially the same as those used in Yabe & Taga [4], were horizontal sine wave gratings drawn with four-cycle luminance. The stimulus image was repainted ten times per second. At the time of repainting, the grating was shifted by a constant distance. The magnitude of the shift, which affects the perceived direction of the gratings, was chosen randomly on a trial-by-trial basis from 11 varieties between 122.4 deg and 237.6 deg. When the magnitude of the shift was equal to half the width of one cycle of the gratings (180 deg), the physical direction of motion was bidirectional (a counterphase grating). Other than the counterphase grating, there were five upward ones that shifted less than 180 deg and five downward ones that shifted more than 180 deg.

The stimuli were presented within a square window of side 18.9 cm on a uniform black background. The visual angle and luminance varied depending on each participant's stationary position on the treadmill and height. The visual angle was approximately 6.0 deg for a participant of average height (165.6 cm). It was approximately 6.7 deg for one of minimum height (156 cm) and 5.6 deg for one of maximum height (175 cm). The magnitude of the counterphase grating shift was 2.36 cm, approximately 0.7 deg when the visual angle of the stimulus window was 5.5 deg. A counterphase grating was presented with the temporal frequency of 5 Hz, and its apparent speed was approximately 7.0 deg/s. The maximum value of the luminance was approximately 31.2 Cd/m^2^ and the minimum was approximately 3.0 Cd/ m^2^; the luminance contrast was approximately 82.6%.

#### Analysis

The participant responses are considered to reflect the physical bias of the grating phase shift and the effect of each condition. When the raw data are fitted with a psychometric function, the sigmoid curve for the probability of downward response may shift both vertically and horizontally. To analyze the vertical shift under each condition, the ratio of the “downward” response (RDR) was calculated for each participant. We regarded individual RDRs as random effects and performed one-way ANOVA with factors of experimental conditions (W, Str, and Sfl). To analyze the horizontal shift of the sigmoid curve, we obtained the point of subjective equality (PSE) as the thresholds for “downward” responses. The PSE was calculated from the probabilities of the “downward” responses, which were calculated against grating phase shift and fitted with a logistic function,



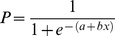
(1).

Free parameters *a* and *b* were estimated by Newton's method. From the fitted functions, PSE, at which the probability of the “downward” responses, *P* in equation (1), equals 0.50, was estimated from the inverse of the function. PSEs were statistically analyzed using one-way ANOVA with factors of experimental conditions (W, Str, and Sfl). All analyses were conducted in R Version 2.10.1. The function made by Prof. Shigenobu Aoki (http://aoki2.si.gunma-u.ac.jp/R/src/logistic_regression.R) was employed for the use of Newton's method for parameter estimation.

### Results and Discussion

**Figure 4:**
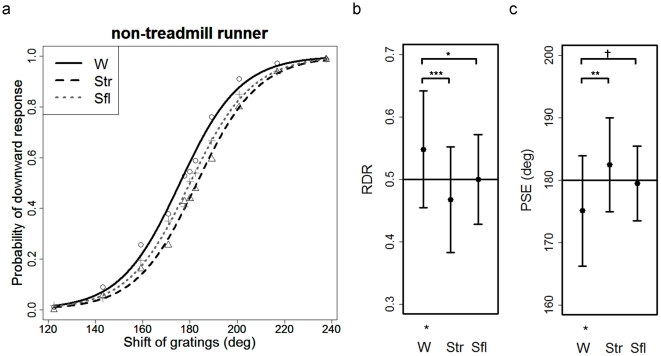
Results of Exp. 1 with non-treadmill runners (nTRs). (**a**) Probability that the nTRs perceived the direction of motion of the stimulus as “downward” is plotted against the shift of the gratings. Circles, crosses, and triangles show the response under Conditions W, Str, and Sfl, respectively. The probabilities of “downward” responses as a function of the grating shift under each condition are fitted with a psychometric function. The solid, dashed, and dotted lines show the fitted curve under Conditions W, Str, and Sfl, respectively. (**b**) Mean ratio of “downward” response (RDR) under each condition. Error bars indicate standard deviation (SD). Asterisks on the top represent a significant difference between the conditions (* *p*<.05 and *** *p*<.001). Asterisk on the bottom represents a significant difference from the chance level (*p*<.05). (**c**) Mean point of subjective equality (PSE) under each condition. Error bars indicate SD. Asterisks and a dagger on the top represent significant and marginally significant differences between the conditions, respectively (** *p*<.01 and *† p* = .04). Asterisk on the bottom represents a significant difference from the chance level (*p*<.05).

For each participant, the relative probability of “downward” motion perception calculated from 10 repetitions was obtained for each of the 11 grating phase shifts under each condition. The averaged data for 18 participants were plotted as a function against phase shift and fitted with a logistic function for each condition (Figure 4a). The more frequently participants reported “downward,” the more the curve shifted to the left. The participants' average RDRs were 0.55 (SD = 0.09), 0.47 (SD = 0.08), and 0.50 (SD = 0.07) under Conditions W, Str, and Sfl, respectively (Figure 4b). Only under Condition W, the RDR was significantly larger than the chance level: 0.50 (two-tailed *t*(17) = 2.182, *p*<.05). One-way ANOVA showed that the effect of the condition was significant (*F*(2,34) = 7.292, *p*<.01). Ryan's method performed as a post hoc analysis showed that the RDR under Condition W was greater than that under Conditions Str (*p*<.001) and Sfl (*p*<.05).

PSE was obtained under each condition for each participant. The participants' average PSE was 175.06 deg (SD = 8.58), 182.44 deg (SD = 7.31), and 179.43 deg (SD = 5.8) under Conditions W, Str, and Sfl, respectively (Figure 4c). Only under Condition W, the PSE was significantly smaller than 180 deg (two-tailed *t*(17) = 2.373, *p*<.05). The one-way ANOVA revealed a significant effect of condition (*F*(2,34) = 6.239, *p*<.01). A post hoc test showed that PSE under Condition W was smaller than that under Condition Str (*p*<.01) and marginally smaller than that under Condition Sfl (*p* = .04). Note that the average slope of the psychometric function at PSE was 0.025 (SD = 0.010), 0.024 (SD = 0.009), and 0.027 (SD = 0.015) under Conditions W, Str, and Sfl, respectively. A one-way ANOVA revealed no significant difference among the conditions.

Consistent with the reports of Yabe & Taga [5], the nTRs showed the effects of treadmill capture (RDR_W>RDR_Str, PSE_W<PSE_Str). In addition, the nTRs did not perceive downward motion in the ambiguous apparent motion display presented on the surface under their feet as they stood, regardless of whether or not they were on the treadmill (RDR_Str = RDR_Sfl = 0.50, PSE_Str = PSE_Sfl = 180). This result supports Hypothesis 1, as shown in the upper part of Figure 1b. This implies that not the bare awareness of the context of being on a treadmill but the ongoing activity of walking biases the perceived direction of visual motion in accordance with the direction of the ground optic flow during natural walking. For the nTRs, the linkage between forward walking and backward optic flow, which they experience daily, is supposed to be a cue to the biased perception of the ambiguous motion direction. Note that these effects cannot be accounted by difference of cognitive loads or head movements among the conditions, since there is no difference in the slope of the psychometric function that reflects sensitivity to visual stimuli.

## Experiment 2

### Methods

#### Ethics Statement

Ethical approval was obtained for this study from the ethical committee of the Graduate School of Education, University of Tokyo, and informed consent was obtained from all participants prior to the initiation of the experiments.

#### Participants

Twenty-two healthy treadmill runners (TRs; 12 males and 10 females; mean age 23.4 years) participated in Exp. 2. The criteria for their selection were that they had exercised on a treadmill at least one day per week for at least 30 min per day in the 12 months prior to participating in our study. The participants completed the questionnaire before the experiments. The mean number of days they had exercised per week was 1.8 (minimum 1, maximum 4), while that per day was 46.1 (minimum 30, maximum 90) min. The TR group had been using the treadmill for a mean of 23.9 (minimum 12, maximum 72) months. They were all naive to the purpose of the study. Each participant had normal or corrected-to-normal vision. We limited the participant height to 180 cm for the same reason given in Exp. 1. Ethical approval was obtained for this study from the ethical committee of the Graduate School of Education, University of Tokyo, and informed consent was obtained from all participants prior to the initiation of the experiments. Data from three participants were excluded from the analysis because the participants reported that they had fixated on the outline of the stimulus, although we had instructed them not to do so. We also excluded data from a participant whose R-squared of the psychometric function was very low (0.03) under Condition Str.

#### Apparatus, procedure, stimuli, and analysis

The apparatus, procedure, stimuli, and analysis were the same as those used in Exp. 1. The visual angle and luminance varied depending on each participant's stationary position on the treadmill and height. The visual angle was 6.0 deg or less for a participant of average height (164.6 cm), 7.0 deg or less for one of minimum height (149 cm), and 5.5 deg or less for one of maximum height (177 cm). The maximum value of luminance was approximately 32.6 Cd/m^2^ and the minimum was approximately 3.2 Cd/m^2^; the luminance contrast was approximately 82.4%.

### Results and Discussion

**Figure 5:**
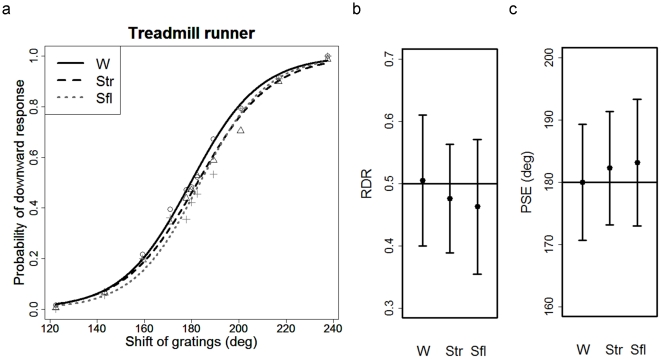
Results of Exp. 2 with treadmill runners (TRs). (**a**) Probability that the TRs perceived the direction of motion of the stimulus as “downward” is plotted against the grating shift. The probabilities of “downward” responses as a function of the shift of the gratings under each condition are fitted with a psychometric function. See the caption of Figure 4a for a description of the symbols and lines. (**b**) Mean RDR under each condition. Error bars indicate SD. (**c**) Mean PSE under each condition. Error bars indicate SD.

The probability that the TRs perceived the direction of motion of the stimulus as “downward” is plotted against the grating shift (Figure 5a). The plot shows that all the conditions have little effect on the perceived direction of visual motion. The average RDR was 0.51 (SD = 0.10), 0.48 (SD = 0.09), and 0.46 (SD = 0.11) under Conditions W, Str, and Sfl, respectively (Figure 5b). The RDR was not significantly different from the chance level: 0.50 under all conditions. An ANOVA showed no significant effect of the condition. The average PSE was 179.95 deg (SD = 9.04), 182.26 deg (SD = 8.85), and 183.13 deg (SD = 9.90) under Conditions W, Str, and Sfl, respectively (Figure 5c). The PSE was not significantly different from 180 deg under all conditions. A one-way ANOVA revealed no significant difference among the conditions. Note that the average slope of the psychometric function at PSE was 0.023 (SD = 0.010), 0.019 (SD = 0.006), and 0.024 (SD = 0.012) under Conditions W, Str, and Sfl, respectively. A one-way ANOVA revealed no significant difference among the conditions.

The results of Exp. 2 demonstrate no “downward” bias under Condition W. This suggests that the link between forward locomotion and backward optic flow on a treadmill, which was likely to bias the perceived direction of motion for the nTRs, was not used by the TRs. In addition, the lack of downward bias under both the standing conditions, regardless of the spatial context, suggests that the expectation of backward optic flow of the treadmill belt, which may be formed by habitual training on it, has no effect on the perceived direction. It is possible that adaptation to treadmill walking, which provides no self-motion in reference to the ground, may generate a new linkage between walking and no optic flow in the TRs.

#### Comparison between Experiment 1 and 2

RDRs were submitted to a 3 (Condition)×2 (Experience) mixed-model ANOVA. The main effect for Condition exerted a significant effect in the analysis (F(2,68) = 6.53, p<.005). No significant main effect of Experience was shown (F(1,34) = .95, p = .34). Although the interaction between Conditions and Experience was not significant (F(2,68) = 1.514, p = .23), we tested the simple main effect of them to learn tendencies of results. There was simple main effect of Condition on nTRs (F(2, 68) = 6.26, p<.005). Ryan's multiple comparisons for Condition (MSe = .005) on nTRs revealed that the RDRs under Condition W were larger than those under Condition Str (p<.001) and Sfl (p<.05).

PSEs were also submitted to a 3 (Condition)×2 (Experience) mixed-model ANOVA. The main effect of Condition exerted a significant effect in the analysis (F(2,68) = 4.95, p<.01). No significant main effect of the Experience was shown (F(1,34) = 1.66, p = .21) in PSEs too. Although the interaction between Conditions and Experience was not significant (F(2,68) = 1.34, p = .27), we tested the simple main effect of them similar to RDRs. There was simple main effect of Condition on nTRs (F(2, 68) = 5.26, p<.01). Ryan's multiple comparisons for Condition (MSe = 47.12) on nTRs revealed that the PSEs under Condition W were smaller than those under Condition Str (p<.005) and marginally smaller than Sfl (p = .06). There was marginal simple main effect of Experience on Condition W (F(1, 102) = 214.83, p = .09) showing that the PSEs was marginally smaller in nTRs than in TRs.

## Discussion

The present experiments clarified the influence of long-term adaptation of treadmill running or walking on treadmill capture. The participants were required to judge whether the apparent motion display of horizontal gratings in front of their feet moved upward or downward. The results of Exp. 1 with nTRs showed that the downward bias to the perceived direction of the stimulus display was observed only under Condition W. This finding supports Hypothesis 1, as shown in the upper part of Figure 1b, which argues that the ongoing activity of walking on a treadmill but not the awareness of being on it biases the perceived direction of visual motion shown in front of the participant's feet in accordance with the direction of the backward optic flow of the ground surface during normal walking on the ground. In Exp. 2 with TRs, no bias to the perceived direction of the stimulus display was observed under the three conditions. This finding also supports Hypothesis 1, as shown in the upper part of Figure 1c, which argues that neither the ongoing activity of walking on a treadmill nor the contextual awareness of being on it biases the perceived direction of visual motion, probably because of adaptation to the linkage between treadmill walking and no self-motion in reference to the ground. Although the direct comparison between Exp.1 and 2 showed that the main effect of experience was not significant, the inference obtained within paradigm of Condition in each experiment should reflect the nature of the difference of effect of the treadmill capture. We can rule out the possibility that these results were influenced by the difference of cognitive load or head movements, because the slopes of the psychometric functions, which should reflect sensitivity to visual stimuli, were not different between conditions in both experiments.

The important point of our research is to clarify the mechanism underlying treadmill capture by examining whether the awareness of being on a treadmill is sufficient to bias the visual motion perception. In relation to this issue, Wright et al. [18] showed that the knowledge of spatial and physical context influences visually induced self-motion perception in an immersive virtual environment. On the other hand, Fukui et al. [19] showed that even if a person is aware that an escalator is stopped, the escalator-specific motor program emerges while stepping onto the escalator. In our study, nTRs showed the absence of downward bias to the perceived direction of motion under Condition Str (Exp. 1). This suggests that the awareness of being on a moving treadmill under this condition did not affect motion perception. It further implies that the treadmill capture phenomenon observed in nTRs is ascribable to the automatic process to perform locomotion, consistent with the broken escalator phenomenon [19]. On the other hand, TRs (Exp. 2) also showed no downward bias to the perceived direction of motion under Condition Str. This result did not support Hypothesis 2, which argues that the awareness of being on a treadmill can induce the downward bias of visual motion direction under Condition Str. It further suggests that TRs provided no evidence to show that contextual awareness alone can activate a possible habitual linkage between walking and the perception of backward optic flow of the treadmill belt. Note that, given the assumption that both nTRs and TRs were aware of the spatial context of being on a treadmill, the difference in the visual perception under Condition W between them implies that the awareness of the spatial context may play an important role in adopting the motor-perceptual linkage to affect visual motion perception depending on the participants' prior experience with treadmill exercise. Although we have argued that participants should have the awareness of being on a treadmill under Conditions W and Str, the difference between Conditions W and Str do not dissociate motor act of walking v.s. standing still and the awareness of the motor acts. Thus, the treadmill capture under Condition W can be affected not only by purely motor act of walking but also by cognitive process such as awareness of the action.

The different results between nTRs and TRs in the present study should provide insight into what type of locomotor-visual adaptation takes place through long-term treadmill exercise. The downward bias to the perceived direction of visual motion observed under Condition W in the nTRs can be attributed to the prior locomotor-visual linkage between walking and backward optic flow of the ground surface in the daily experience of locomotion. This implies that although nTRs should be aware of walking on a treadmill, the ongoing walking movements may implicitly induce the use of the prior locomotor-visual linkage when nTRs walk on the ground. In contrast, the absence of downward bias to the same stimuli in the TRs can be attributed to the treadmill-specific motor-perceptual linkage between walking and no optic flow. The latter linkage is likely to be acquired through the long habit of using a treadmill. In fact, Bruggeman et al. [20] reported that one can acquire a new type of locomotor-visual linkage in novel environments through the process of adaptation based on optic flow.

Although the present study did not directly observe the process whereby the treadmill-specific motor-perceptual linkage is acquired, it is important to understand how the implied linkage is generated. Walking in a novel environment may generally cause a conflict between the execution of body movements and the expectation of visual feedback. To eliminate the conflict, people may recalibrate the locomotor-visual linkage, whose stability can be estimated by the persistency of the aftereffects that are observed when they return to a familiar environment. For example, Pelah and Barlow [3] showed that after people perform more than 10 min of treadmill exercise, they experience a sensation of apparently moving at a markedly accelerated pace, which lasts only 2–3 min and then disappears. In contrast to this single experience of recalibration and washout of the novel locomotor-visual linkage within a short period, TRs in the present study may experience numerous repetitions of recalibration during treadmill walking and washout during normal walking in their daily life. This suggests that the implied locomotor-visual linkage that affects visual motion perception in TRs might be established through multiple experiences with the treadmill over a longer time.

The treadmill capture represents the perceptual bias to the direction of ambiguous apparent motion in accordance with the direction of optic flow, which we experience on a daily basis. In contrast, Durgin et al. [21]–[22] reported another type effect in which the perceived speed of optic flow was reduced during walking. One interpretation is that we have two distinct types of adaptation system: one which leads to the bias with experience-mediated compensation, as shown in the treadmill capture, and one which leads to the bias with a repulsive aftereffect, as shown in the reduction of perceived visual speed while walking. Durgin et al. [21]–[22] explained the reduction of perceived speed by extending Barlow's [23] sensory inhibition theory: highly correlated events produce shifts in coding strategies that take advantage of the redundancy by strengthening inhibitory connections. Durgin et al. [21]–[22] considered that the speed of optic flow is reduced when it is predictable by multiple signals during walking. Although whether the type of influence of experience on perception occurs depends on the perceptual tasks, both mechanisms must contribute to the robust perception of the complex environment.

### Conclusion

In the experiments in the present study, the participants had to resolve two ill-posed problems. The first is the correspondence problem that occurs during the perception of apparent motion. To resolve it, the visual system has to compensate for the lack of information on the correspondence of consecutive retinal images. A crucial point of treadmill capture observed in nTRs is that the information on the ongoing activity of locomotion was used as a constraint to determine the perceived direction of visual motion. Second, locomotion in an environment exposes a person to the ill-posed problem in motion perception: retinal images cannot provide a unique motion perception because of the dynamic relationship between self and the environment. To solve this problem, one has to have a constraint to determine this relationship. The present study suggests that nTRs and TRs have different implicit assumptions during treadmill walking: nTRs assume that they are traveling in an environment, whereas TRs assume that they are not traveling in an environment despite performing walking movements. An important implication of this study is that the long-term experience of body movements in a novel environment alters the way the body movements influence visual perception, which should lead to the generation of adaptive behaviors in a dynamic environment.
